# A collaborative editorial model supports research integrity

**DOI:** 10.1186/s12916-022-02565-0

**Published:** 2022-09-21

**Authors:** BMC Medicine

**Affiliations:** The Campus, 4 Crinan Street, London, UK

Research is integral to every aspect of our lives - from the development of vaccines to the implementation of Government policy, our understanding of climate change to the impact of our actions on society. The past two years have clearly brought to the fore the importance and impact of trust in science and research. Research and development of COVID-19 vaccines and therapies has been conducted both quickly and safely, going through a peer review process whereby independent experts in relevant research areas assess the quality of research before publication. The integrity, accuracy and reliability of this research needs to be as accurate and trustworthy as it can be. We remain committed to working with researchers to uphold the validity of academic research - responsible conduct, peer review and best practice is critical to this. Improving reproducibility and research integrity in scientific publishing, aided by the peer review process, influences the public's trust of research and will ultimately affect the adherence to and credibility of medical guidance/guidelines derived from it.

Fittingly, research integrity is the theme of this year’s Peer Review week (Sept 19th-23rd). This is a global annual event that celebrates the essential role that peer review plays in maintaining research quality. In this Editorial, we provide insight into how we aim to maintain research integrity during our review process.

The foundations underlying research integrity are defined by the following principles, allowing others to have confidence and trust in the methods and the findings of the research adhering to them:

- Rigor
- Honesty
- Transparency
- Accountability
- Adherence to appropriate ethical, legal and professional frameworks

Of course, authors bear most of the responsibility for research integrity; however, other stakeholders in the process from research submission to publication also play a big role. The opportunity for peers to scrutinize a colleague's research during the peer review process may highlight, for instance, a lack of transparency in the methods or a lack of rigor within the analyses. Editorial policies at different journals also encourage (or mandate for some data types) deposition of data underlying conclusions, and adherence to relevant reporting guidelines, ensuring transparency and author accountability.

At *BMC Medicine,* in-house editors, in addition to handling papers directly, also work closely with Editorial Board Members (EBMs) to manage the peer review process: they assess submissions, find and invite appropriate reviewers for those manuscripts we decide are appropriate to consider further, ensure reviewers return useful reports in a timely manner, and then make editorial decisions based on those reports. Further, we ensure that research integrity is maintained according to our editorial policies.

Peer review can greatly improve research integrity, and working with our EBMs is an essential part of this process. Robust peer review underpins the scientific publishing process, and readers should be confident that a published research paper has been scrutinized by a minimum of two independent reviewers who are experts in their fields. Involving academic EBMs in this process provides an additional opportunity for expert involvement. In order to ensure proper guidance from the professional in-house editors and adequate safety mechanisms, we adhere to the following practices.

All editors, including EBMs, are asked to adhere to Springer Nature’s Code of Conduct that sets out minimum standards of manuscript handling and peer review. In addition, the EBM would have a specific in-house editor to contact for any manuscript they have been assigned, who provides continuous support and feedback during the peer review process. Further, Springer Nature provides a number of courses for editors that focus on research integrity,

In order to ensure that we’re adhering to peer review standards, all intermediate decisions are checked by in-house editors and all final decisions are checked by the Chief Editor. We ensure that the reviewers are suitably qualified to review the manuscript, that the reviewers’ comments are fair, and the authors implement the reviewers’ and editors’ suggestions to strengthen the paper prior to publication, if appropriate.

Importantly, during this process, we also screen manuscripts for adherence to appropriate ethical guidelines. For instance, any research involving human participants, data or material must have been approved by an appropriate ethics committee and have been conducted in accordance with the Declaration of Helsinki, the general principles of which put the patient first and ensure that the research adheres to ethical standards that promote and ensure respect for all human subjects, while protecting their health and rights. We also mandate informed consent from all human participants (or their parent or legal guardian in the case of children under 16). Clinical trials, which prospectively test the effects of an intervention, are required to be registered on a suitable publically accessible registry. Trial registration reduces duplication of research efforts, improves awareness of trials for clinicians, researchers, patients and the public. In addition to research focusing on human participants and data, we also consider translational research which may involve animal subjects. Research involving animal experimentation must comply with institutional, national, or international guidelines, and must be approved by an appropriate ethics committee, details of which should be included in the manuscript. Further, we advocate complete and transparent reporting of biomedical and biological research. To this end, we encourage or mandate the use of various reporting checklists and guidelines for authors to ensure adequate standards of reporting according to the type of research.

If we do encounter complex research integrity issues, we have a dedicated in-house Research Integrity Team that supports editors to resolve publication ethics issues in a COPE (Committee on Publication Ethics)-compliant manner.

Collaboration in the processes that aid research integrity is paramount to maintain a resilient system of trust within scientific research. The partnership between in-house editors and EBMs provides the EBMs with editorial experience and a ‘behind the scenes’ insight, while simultaneously expanding the in-house editorial team’s specialist knowledge and increasing our engagement with the community. Editors, both in-house and EBMs, work hard to ensure robust and resilient editorial processes in order to maintain trust in the published research. Many eyes check each scientific manuscript that we consider, so there is a cumulative effect ensuring that we publish robust and valid science. The EBMs’ day-to-day editorial work contributes to greater community transparency in this process, thereby further strengthening research integrity.

At a time when trust and reliability in the guidance of medical experts is easily questioned and dismissed, the integrity of medical research has never been so important. By focusing our editorial processes to ensure that only the most robust research is published, the editors at *BMC Medicine* aim to aid the healthcare community and to ultimately benefit the community at large.

